# Identification of metabolites from complex mixtures by 3D correlation of ^1^H NMR, MS and LC data using the SCORE-metabolite-ID approach

**DOI:** 10.1038/s41598-023-43056-3

**Published:** 2023-09-22

**Authors:** Stephanie Watermann, Marie-Christin Bode, Thomas Hackl

**Affiliations:** 1 Institute of Organic Chemistry, University of Hamburg, Martin-Luther-King-Platz 6, 20146 Hamburg, Germany; 2 Hamburg School of Food Science – Institute of Food Chemistry, University of Hamburg, Grindelallee 117, 20146 Hamburg, Germany

**Keywords:** Metabolomics, NMR spectroscopy

## Abstract

Not only in metabolomics studies, but also in natural product chemistry, reliable identification of metabolites usually requires laborious steps of isolation and purification and remains a bottleneck in many studies. Direct metabolite identification from a complex mixture without individual isolation is therefore a preferred approach, but due to the large number of metabolites present in natural products, this approach is often hampered by signal overlap in the respective ^1^H NMR spectra. This paper presents a method for the three-dimensional mathematical correlation of NMR with MS data over the third dimension of the time course of a chromatographic fractionation. The MATLAB application SCORE-metabolite-ID (*Semi-automatic COrrelation analysis for REliable metabolite IDentification*) provides semi-automatic detection of correlated NMR and MS data, allowing NMR signals to be related to associated mass-to-charge ratios from ESI mass spectra. This approach enables fast and reliable dereplication of known metabolites and facilitates the dynamic analysis for the identification of unknown compounds in any complex mixture. The strategy was validated using an artificial mixture and further tested on a polar extract of a pine nut sample. Straightforward identification of 40 metabolites could be shown, including the identification of *β*-d-glucopyranosyl-1-*N*-indole-3-acetyl-*N*-l-aspartic acid (**1**) and *N*^α^-(2-hydroxy-2-carboxymethylsuccinyl)-l-arginine (**2**), the latter being identified in a food sample for the first time.

## Introduction

Both in the field of natural product chemistry and in metabolomics studies, the unambiguous identification of chemical compounds is of great importance. Especially the identification of potential biomarker metabolites is of outstanding interest e.g. for disease diagnosis and prediction as well as for food authentication^1–4^.

Identification of small-molecules both in synthetic chemistry, in natural product chemistry and metabolomics is mainly achieved by means of *Nuclear Magnetic Resonance Spectroscopy* (NMR) and/or *Mass Spectrometry* (MS), both of which are as well the leading analytical methods in metabolomics^1,5–10^. NMR spectroscopy is a highly reproducible method requiring only minimal sample preparation and the spectra additionally provide detailed structure information^2,6,8,10,11^. MS or hyphenated methods such as MS coupled to *gas chromatography* (GC) or *liquid chromatography* (LC) show clear benefits regarding the high sensitivity as well as the possibility to calculate the chemical formula of the molecule of interest in the case of high-resolution mass spectrometers^2,6,7,12,13^ However, the identification of unknown small-molecules from complex mixtures using either NMR or MS is often not possible without individual isolation. Particularly in the field of NMR based metabolomics studies, the assignment of specific NMR signals is often based on database comparison. In addition to some inherent errors that may occur in any database, experimental conditions such as solvent, pH, or ionic strength of the sample have a tremendous impact on the chemical shift, making comparison and exclusive use of data from a database difficult and can lead to unreliable assignments^14^. Therefore, a requirement for reliable identification is the use of at least two orthogonal, independent analytical methods^15^. Recently published methods based on the powerful molecular network approach, such as the NMR-based SMART (Small Molecule Accurate Recognition Technology) method and the LC–MS/MS-based GNPS (Global Natural Products Social) method, recognize similar structural motifs and can identify a variety of structures based on database spectra. However, for new natural products, isolation is usually necessary for adequate characterization^16,17^. Isolation and purification often requires the time- and resource-intensive development of an individual isolation strategy for each compound of interest^18–20^.

Thus, direct and simultaneous identification of multiple compounds from a complex mixture is a worthwhile endeavor. As NMR and MS are complementary methods providing supplementary data of the compounds of interest, a combination of both analytical platforms for facilitated identification is a promising method^7,9,10,21^. Furthermore, the required condition of two independent analytical platforms for reliable identification of known or unknown metabolites is met simultaneously^15,22^.

Various approaches exist for combining NMR and MS, e.g. hardware-based, cheminformatics-based or statistics-based^1^. The hardware-based combination of MS and NMR, such as online LC–MS–NMR, bears some difficulties in practice, for instance, the need to use deuterated solvents for LC, different sample requirements and general technical demands^2^. An example for a cheminformatic combination of NMR and MS is the recently published NMR/MS translator and the method called SUMMIT MS/NMR^1,5,19^. The NMR/MS translator method relies on a detection of possible metabolites by NMR database query and following calculation and comparison of expected *m/z* ratios in mass spectra^5^. Therefore, this method is only useful for the identification of already known metabolites^5^. The SUMMIT MS/NMR method on the other hand can also be used for the identification of unknown metabolites^19^. Exact masses from MS spectra are used for calculation of possible molecular formulas which are then translated into all possible structures^19^. Prediction and comparison of NMR spectra of all possible structures with experimental NMR spectra then leads to straightforward identification of known and unknown metabolites^19^. A further development of SUMMIT MS/NMR is the SUMMIT Motif approach, which is based on determination of molecular structural motifs (MSMs) by identification of ^1^H and ^13^C NMR spin systems and subsequent database query, either consisting of experimental NMR data (COLMAR MSM Metabolomics Database (MDB)) or of empirically predicted chemical shift data (pNMR MSMMDB)^23^. Statistics-based correlation of NMR and MS data can be achieved by *Statistical heterospectroscopy* (SHY), which is an analogous approach to the *statistical total correlation spectroscopy* (STOCSY), correlating NMR data^24,25^. Both methods enable the detection of correlated signals due to actual structural connectivity or intermolecular correlations resulting from the connectivity of biological metabolic pathways^24,25^. However, using statistics-based approaches such as SHY, large sample sets are required for statistical analysis and no distinction can be made between the type of correlation.

Correlation of NMR and MS data can also be achieved after incomplete separation of compounds by liquid chromatography^26–29^. An example is the method called NMR/LC–MS *parallel dynamic spectroscopy* (NMR/LC–MS PDS), introduced by Dai et al*.*, which can be used for the manual correlation by visualization of NMR and LC–MS data of the different fractions^26,27^. The method called *three-dimensional cross correlation* (3DCC) by Behnken *et al.* enables the mathematical correlation of NMR and LC–MS data and was used for the structure elucidation of glycan mixtures^28,29^. However, in contrast to the identification of glycans using the structural reporter group concept, the structure elucidation of metabolites of different compound classes requires information on all NMR signals of the molecule^29,30^. Therefore, we present here the SCORE-metabolite-ID (*Semi-automatic COrrelation analysis for REliable metabolite IDentification*) method as a generally applicable technique for the mathematical correlation of NMR and DI-MS (*direct injection* MS) data after incomplete separation by (flash) chromatography to facilitate reliable identification of known and unknown metabolites of several compound classes from a complex mixture without individual isolation. The developed MATLAB app *SCORE-metabolite-ID* is not limited to specific classes of molecules and allows not only the calculation of correlation coefficients for specific signals, but also the detection of highly correlated NMR or MS signals in a semi-automatic way. Thereby, associated NMR signals from complex mixture sample can be assigned to specific mass-to-charge ratios, which on the one hand saves time of individual isolation and, more importantly, leads to more reliable identification since two orthogonal, independent analytical methods are used simultaneously.

## Results and discussion

### General concept

The method for the correlation of NMR and MS signals is based on the third dimension of liquid chromatographic separation. After liquid chromatography, NMR and DI–MS spectra are acquired from each fraction. The distribution of any one analyte among multiple fractions, intended by the column’s separation capability and choice of fractionation conditions, is desired because it leads to NMR and DI–MS signals of the same compounds in several consecutive fractions, depending on the respective elution window. The *two-dimensional* (2D) representation of a NMR signal at a specific chemical shift value against the time-domain, i.e. fraction numbers, can be extracted easily and is called *extracted delta chromatogram* (EDC), following the already introduced 3DCC method^28^. Accordingly, the 2D plot of a specific *m/z* value from DI–MS spectra against the successive fractions is called *extracted mass chromatogram* (EMC). The different compounds in the mixture have different elution times with different elution profiles. However, the signals of the compounds in the NMR and MS spectra, i.e. in the EDCs and EMCs, each show the same elution profile. To produce proper EDCs and EMCs, appropriate acquisition and processing parameters need to be used, to diminish changes in chemical shift resp. *m/z* value across the different fractions. The generated EDCs and EMCs can be correlated using Pearson Correlation leading to the detection of related NMR and MS signals. The SCORE-metabolite-ID app can be used in a semi-automatic way, i.e. after manual selection of a specific EDC (or EMC) all EMCs (or EDCs) in each of the selected fractions are calculated automatically. The *m/z* values or chemical shifts of EMCs and EDCs with a correlation coefficient of e.g. > 0.95 can then be displayed together with the respective intensity values. This enables the detection of highly correlating EMCs and EDCs in a semi-automatic way, facilitating the identification of metabolites without their individual isolation.

### Proof of concept using artificial mixture

**Figure 1:**
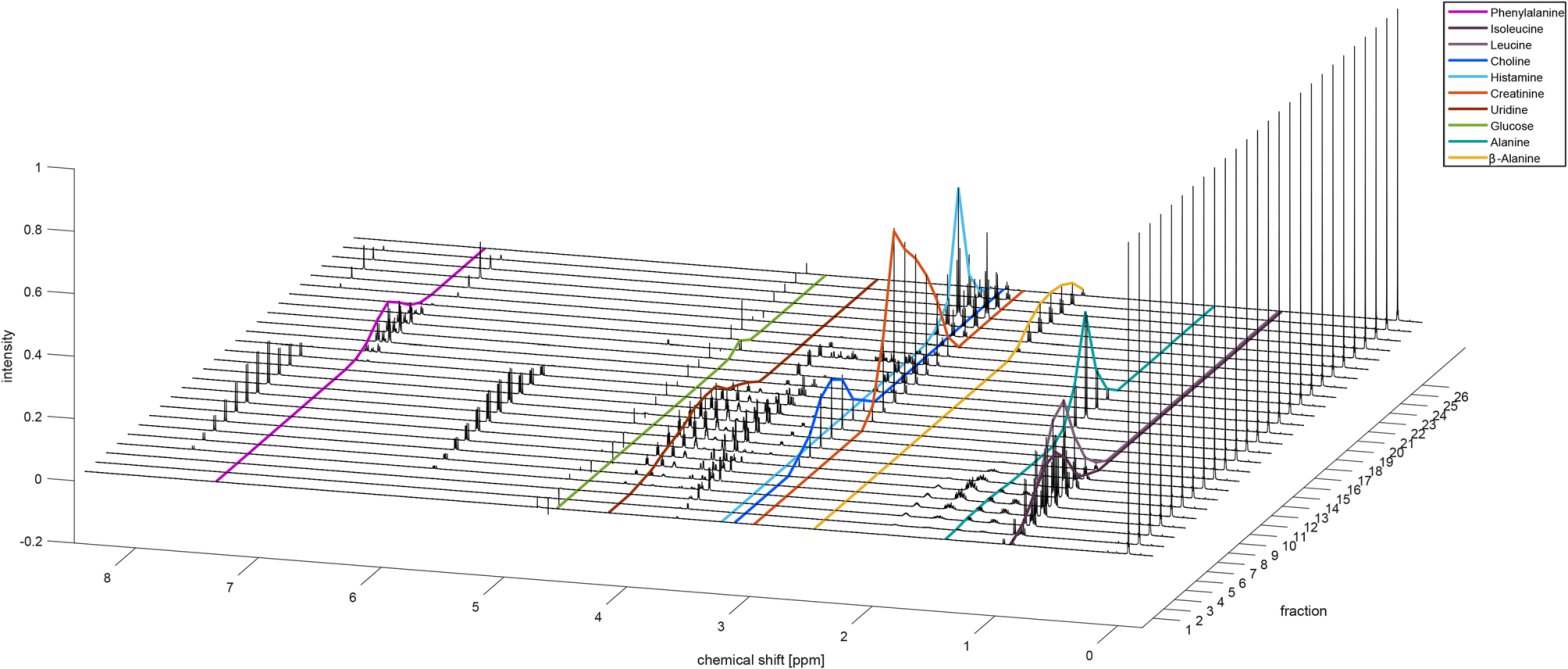
NMR Spectra of the artificial mixture. An EDC for each of the ten different compounds is highlighted in different colors.

The general concept of the method was investigated using an artificial mixture containing a total of ten compounds, each in different concentrations, divided into 26 artificial fractions. The samples were prepared as stated in the experimental section and as shown in Table S1 in the Supporting Information. NMR and DI–MS spectra of each fraction were acquired. To resemble a real metabolite mixture, compounds of different substance classes were added in varying concentrations over a different number of samples corresponding to different elution windows in a real fractionation. Furthermore, compounds causing signals at similar chemical shifts as well as isobaric compounds such as leucine and isoleucine or alanine and *β*-alanine are used. Figure 1 shows the NMR spectra of the 26 samples. The corresponding DI-MS spectra in positive ionization mode are shown in the Supporting Information (Fig. S1).

**Figure 2:**
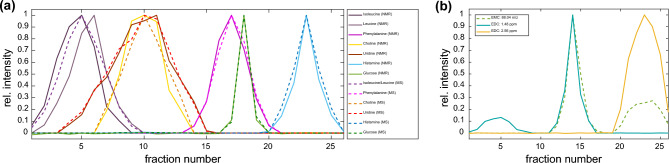
(**a**) Two-dimensional representation of the normalized NMR and MS signals (i.e. EDC and EMC) of exemplary compounds in the artificial mixture over the 26 fractions. (**b**) EMC of the isobaric amino acids alanine and *β*-alanine at *m/z* of 88.04 ([M-H]^-^) together with EDCs of alanine (1.48 ppm) and *β*-alanine (2.56 ppm).

Both, in the NMR and MS spectra, signal amplitude intensity is proportional to the concentration of the compound. Provided that the same sample preparation is performed and the same measurement parameters are used, signal amplitude profiles over a series of fractions can be extracted. In order to produce adequate EDC end EMC profiles, signals have to be consistent regarding the chemical shift resp. *m/z* value over all fractions. Thus, for NMR spectra a bucketing can be performed within the app to circumvent effects of small shifts due to pH changes, differences in ion strength etc. Furthermore, the use of a suitable buffer (e.g. phosphate buffer in D_2_O) is recommended. For mass spectra, external and internal mass calibration was performed to ensure exact *m/z* values over all fractions. The resulting 2D plot of specific NMR signals (i.e. EDCs) and MS signals (i.e. EMCs) of six exemplary compounds can be used for visualization and is shown in Fig. 2a.

**Figure 3:**
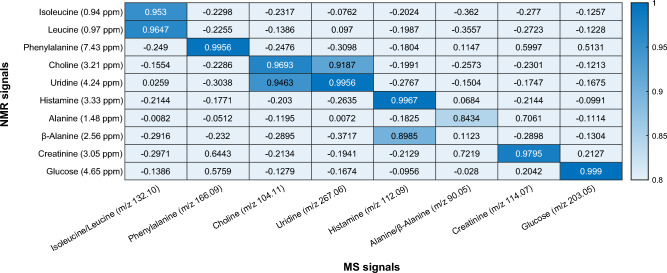
Pearson Correlation Coefficients of NMR and DI-MS signals in positive ionization mode of the artificial mixture over all 26 fractions. The correlation coefficients are highlighted according to the color bar.

Using the SCORE-metabolite-ID app, Pearson correlation coefficients of several EDCs and EMCs can be calculated in a semi-automatic way. The higher the coefficient, the more likely there is a correlation between the respective NMR and MS, or NMR and NMR signal. Figure 3 shows some correlation coefficients between specific EMCs and EDCs of the artificial mixture over all 26 fractions. In general, a correlation coefficient > 0.95 is considered the threshold for a true correlation. In some cases, however, the correlation coefficient may be slightly lower, e.g. for broad signals or signal shifts due to small pH differences. In most cases, an unambiguous correlation with coefficients > 0.98 between EDC and EMC can be observed. The co-eluting compounds choline and uridine as well as histamine and *β*-alanine also show relatively high coefficients between EDCs and EMCs of the respective other compound, but nevertheless the EDCs and EMCs that actually belong together show much higher correlation coefficients. An unambiguous correlation of isobaric compounds such as alanine and *β*-alanine over the total of all 26 fractions is not possible, since the EMC of *m/z* 88.04 ([M-H]^-^) in negative ionization mode shows two maxima, while EDC of *β*-alanine (2.56 ppm) shows one maximum as does alanine (1.48 ppm), which in addition also shows signal intensity in the earlier fractions 1–7, originating from signals of isoleucine at the same chemical shift (cf. Fig. 2b).

**Figure 4:**
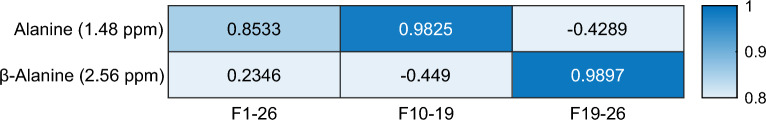
Pearson correlation coefficients of the NMR signals of alanine and *β*-alanine to the MS signal at *m/z* 88.04 ([M–H]^−^ of alanine and *β*-alanine) in the artificial mixture using all fractions 1–26 (column 1) or only a subset of fractions (columns 2 and 3).

However, using the SCORE-metabolite-ID app, it is possible to only select a part of all fractions for the calculation of correlation coefficients in such cases. The calculated Pearson correlation coefficients between the specific EDCs and EMCs of alanine and *β*-alanine using all 26 fractions (column 1) or using only a subset of fractions (columns 2 and 3) are shown in Fig. 4. Thus, clear detection of related EMCs and EDCs is possible, even for isobaric compounds. This flexibility of the SCORE-metabolite-ID method is highly important. In Fig. 4 this case is shown for the same *m/z* value of compounds. However, this is also highly important for the 1D ^1^H NMR spectra with a chemical shift dispersion of approx. 10 ppm. This rather small chemical shift dispersion often leads to NMR signals appearing at the same chemical shift but originating from different compounds in different fractions. If correlation were performed only for all fractions obtained, this could negatively affect the calculated correlation coefficient and some results could be missed.

The isobaric amino acids leucine and isoleucine show slightly lower correlation coefficients in Fig. 3 since both compounds show a slightly different co-elution profile. However, the SCORE-metabolite-ID app can also be used to correlate only EDCs with each other. Table S2 in the Supporting Information shows correlation coefficients of all EDCs of leucine and isoleucine and illustrates the unambiguous detection of all NMR signals of both compounds, even for isobaric compounds with similar elution profile. The results of the artificial mixture demonstrate that correlation of NMR and DI-MS signals that show signal intensity in a series of fractions (e.g. 3 fractions for glucose or 12 fractions for uridine) is possible and allows the detection of associated NMR and MS signals of the same compound. Furthermore, NMR signals can be correlated with each other which facilitates the detection and identification of isobaric compounds.

### Application to complex pine nut extract

**Figure 5:**
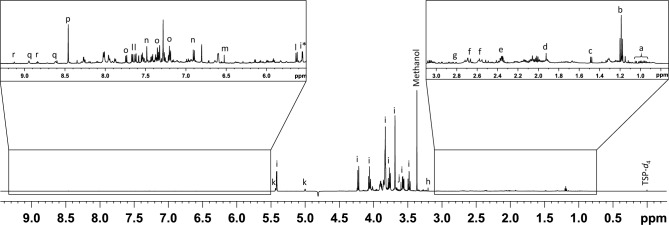
^1^H NMR spectrum of the total polar pine nut extract showing signals from carbohydrates in high concentration, as well as signals in the aliphatic and aromatic region of lower concentration. a: l-isoleucine, l-leucine and l-valine, b: ethanol, c: l-alanine, d: acetic acid, e: l-glutamic acid, f: citric acid, g: l-aspartic acid, h: choline, i: sucrose, j: d-pinitol, k: raffinose, l: IAA-Asp-*N*-Glc (**1**), m: fumaric acid, n: l-tyrosine, o: l-tryptophan, p: formic acid, q: nicotinic acid, r: trigonelline.

After validation using an artificial mixture, the method was used for the identification of metabolites in a polar extract of pine nuts because it is a complex matrix consisting of a large number of different compounds of biological origin. The NMR spectrum of the total extract in Fig. 5 shows many overlapping signals originating from many compounds of different substance classes, such as carbohydrates, organic acids, amino acids, aromatic compounds etc., in large concentration differences. Direct identification of metabolites from the total extract spectrum is therefore hardly possible, especially for the lower concentrated metabolites.

The polar extract was fractionated using an amino-functionalized flash column with acetonitrile and water as eluents under HILIC conditions. ^1^H NMR and DI–ESI–MS spectra in positive and negative ionization mode were acquired from each fraction independently. After data processing, NMR and MS data were imported into MATLAB software using the in-house developed script and analyzed using the SCORE-metabolite-ID app. So far, 40 metabolites could be identified using this method. A comprehensive list of all identified metabolites, such as different carbohydrates, amino acids, nucleosides and nucleotides, organic acids and betaines, including their respective correlation coefficients can be found in Table S3 in the Supporting Information. Among the other compounds, *β*-d-glucopyranosyl-1-*N*-indole-3-acetyl-*N*-l-aspartic acid (IAA-Asp-*N*-Glc) (**1**), as well as a condensation product of arginine and citric acid, *N*^α^-(2-hydroxy-2-carboxymethylsuccinyl)-l-arginine (**2**) were identified in the sample. Compound (**2**) was already identified in bulbs of lilies (*Lilium maximowiczii*) and annual shoots of pear trees in 1983 and 1984, but has not yet been found in any other sample to the best of our knowledge^31,32^. The detailed identification process, including all spectroscopic data of both compounds, is shown below.

#### Identification of (1)

**Figure 6:**
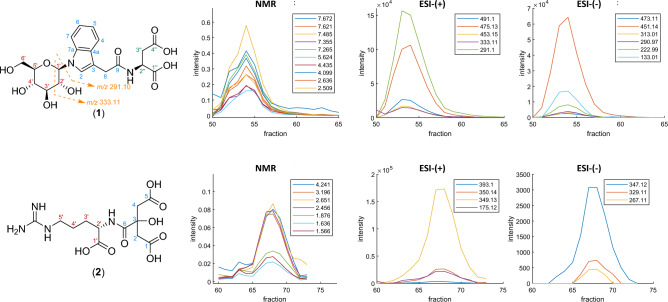
Chemical structures of IAA-Asp-*N*-Glc (**1**) and condensation product of l-arginine and citric acid (**2**) together with 2D plots of EDCs and EMCs in positive and negative ionization mode of (**1**) (upper part) and (**2**) (lower part).

Several NMR signals in the aromatic region of fractions 51–59, each of which showed similar elution profiles, allowed for the straightforward detection of a spin system of an indole derivative. Using the SCORE-metabolite-ID app, automatic detection of EMCs in positive ionization mode with high correlation coefficients to EDC at 7.485 ppm (cf. Table S4), allowed for the calculation of the exact mass of the metabolite of interest and thus for the determination of the most probable molecular formula C_20_H_24_N_2_O_10_ (exact mass: 452.1431 Da). Furthermore, various NMR signals located not only in the aromatic, but also in the aliphatic and carbohydrate region of the NMR spectrum, showed high correlation coefficients (> 0.96) in fractions 51–65 (cf. Table S5). The doublet at 5.63 ppm could be identified as the anomeric proton of a carbohydrate moiety. A selective TOCSY experiment was performed on this well-isolated NMR signal and revealed the presence of glucose (cf. Fig. S2). From the coupling constant of 9.2 Hz, it could be concluded that glucose is present in the *β* configuration. The 2D HMBC experiment confirms the presence of the glucose-*N*-indole bond (cf. Fig. S3). The two NMR signals in the aliphatic region (2.49 ppm and 2.65 ppm) showed coupling to a doublet of a doublet at 4.43 ppm like an ABX spin system which could be identified as l-aspartic acid. The selective TOCSY experiment in Fig. S2 proves the coupling. Furthermore, coupling between indole spin system and two diastereotopic protons at 3.78 ppm and 3.84 ppm could be detected, which led to the conclusion that l-aspartic acid is coupled to indole-3-acetic acid via an amide bond. HMBC coupling between H-2’’ and C-9 could not be detected. However, the NMR spectrum of fraction 55 acquired in a mixture of H_2_O/D_2_O (ratio 9:1) showed an additional doublet at 7.97 ppm (*J* = 7.6 Hz, *I* = 1) originating from the amide proton (cf. Fig. S4). The change in multiplicity of H-2’’ from a doublet of doublet (*J* = 9.3, 4.0 Hz) in the NMR spectrum recorded in D_2_O to a doublet of doublet of doublet (*J* = 4.1, 8.6, 8.7 Hz) in the NMR spectrum acquired in H_2_O/D_2_O (9:1) confirms the assumption that the doublet at 7.97 ppm is indeed caused by the amide proton. Automatic detection of correlating EMCs to EDC at 7.485 ppm (cf. Table S4) additionally revealed *m/z* 291.10 and 333.11 to be highly correlating. The corresponding correlation coefficients to all EDCs are listed in Table S6. Both signals at *m/z* 291.10 and 333.11, the first also being the most intense highly correlating signal, result from the fragmentation of (**1**) as indicated in Fig. 6. This fragmentation pattern is consistent with data published in the literature^33^. ^1^H NMR (600 MHz, D_2_O, pH 7.0, TSP-*d*_4_): *δ* [ppm] = 7.67 (d, ^3^*J* = 8.0 Hz, H-4), 7.63 (d, ^3^*J* = 8.3 Hz, H-7), 7.49 (s, H-2), 7.35 (dd, ^3^*J* = 7.9 Hz, ^3^*J* = 8.1 Hz, H-6), 7.27 (dd, ^3^*J* = 7.9 Hz, ^3^*J* = 7.9 Hz, H-5), 5.63 (d, ^3^*J* = 9.2 Hz, H-1′), 4.43 (dd, ^3^*J* = 9.3 Hz, ^3^*J* = 4.0 Hz, H-2′′), 4.10 (dd, ^3^*J* = 9.2 Hz, ^3^*J* = 9.2 Hz, H-2′), 3.89–3.93 (m, H-6′a), 3.84 (d, ^2^*J* = 16.3 Hz, H-8a), 3.83–3.77 (m, H-6′b), 3.78 (d, ^2^*J* = 16.0 Hz, H-8b), 3.74–3.79 (m, H-5′), 3.73–3.78 (m, H-3′) 3.68 (dd, ^3^*J* = 9.4 Hz, ^3^*J* = 9.4 Hz, H-4′), 2.65 (dd, ^2^*J* = 15.5 Hz, ^3^*J* = 3.9 Hz, H-3′′a), 2.49 (dd, ^2^*J* = 15.6 Hz, ^3^*J* = 9.3 Hz, H-3′′b). ^13^C NMR (150 MHz, D_2_O, pH 7.0, TSP-*d*_4_): *δ* [ppm] = 181.5 (C-4′′, C-1′′), 176.7 (C-9), 139.4 (C-7a), 130.8 (C-4a), 127.2 (C-2), 125.8 (C-6), 123.7 (C-5), 122.2 (C-4), 113.2 (C-7), 112.9 (C-3), 87.1 (C-1′), 80.9 (C-5′), 79.3 (C-3′), 74.4 (C-2′), 72.3 (C-4′), 63.2 (C-6′), 56.1 (C-2′′), 42.6 (C-3′′), 35.0 (C-8). The ^13^C NMR data were obtained from the HSQC and HMBC spectra.

#### Identification of (2)

Compound (**2**) could be identified in fractions 64–70 in the polar extract of the pine nut sample. Two AB spin systems (2.47 and 2.66 ppm, *J* = 15.1 Hz; 2.56 and 2.71 ppm, *J* = 15.2 Hz) were detected. Semi-automatic detection of corresponding EMCs revealed several highly correlating *m/z* values (cf. Table S7), which allowed the calculation of the molecular formula C_12_H_20_N_4_O_8_ (exact mass: 348.1281 Da). Further correlating EDCs at 4.24 ppm (dd, *J* = 4.6, 6.7 Hz), 3.19 ppm (m), 1.88 ppm (m), 1.64 ppm (m) and 1.57 ppm (m) were detected semi-automatically. The calculated correlation coefficients are listed in the Supporting Information (Table S8). Selective and 2D TOCSY experiments confirm the presence of three spin systems (cf. Fig. S5). Considering the molecular formula as well as information from NMR spectra, it could be concluded that compound (**2**) is a condensation product of citric acid and l-arginine, namely *N*^α^-(2-hydroxy-2-carboxymethylsuccinyl)-l-arginine. ^1^H NMR (600 MHz, D_2_O, pH 7.0, TSP-*d*_4_): *δ* [ppm] = 4.24 (dd, ^3^*J* = 6.6 Hz, ^3^*J* = 4.5 Hz, H-2′), 3.16–3.22 (m, H-5′), 2.71 (d, ^2^*J* = 15.2 Hz, H-2a), 2.66 (d, ^2^*J* = 15.1 Hz, H-4a), 2.56 (d, ^2^*J* = 15.2 Hz, H-2b), 2.47 (d, ^2^*J* = 15.1 Hz, H-4b), 1.86–1.90 (m, H-3′a), 1.76–1.81 (m, H-3′b), 1.60–1.65 (m, H-4′a), 1.54–1.60 (m, H-4′b). ^13^C NMR (150 MHz, D_2_O, pH 7.0, TSP-*d*_4_): *δ* [ppm] = 181.1 and 181.2 (C-1 and C-5), 181.1 (C-1′), 179.4 (C-6), 77.6 (C-3), 56.9 (C-2′), 47.4 and 47.5 (C-2 and C-4), 43.4 (C-5′), 31.6 (C-3′), 26.2 (C-4′). The ^13^C NMR data were obtained from the HSQC and HMBC spectra.

## Conclusion

The SCORE-metabolite-ID app presented here is a unique and universally applicable method in the vast field of metabolite identification. The method greatly facilitates identification of known and unknown metabolites from complex mixtures with comparatively little practical effort. The method relies on mathematical correlation of ^1^H NMR and DI–MS signals after liquid chromatography of the sample to distribute the respective analytes across multiple fractions. The app allows the semi-automatic detection of highly correlating EDCs and EMCs, thereby facilitating identification of known and unknown compounds enormously. After fast and reliable dereplication of known metabolites, the focus can be put on the identification of unknown metabolites. The application of the SCORE-metabolite-ID app thus quickly enables the subsequent targeted acquisition of e.g. selective or 2D NMR experiments in a dynamic manner depending on the individual compounds to arrive at a reliable identification, especially for unexpected and unknown metabolites. As with all NMR-based methods, the sensitivity of the SCORE-metabolite-ID approach is mainly limited by the rather low sensitivity of NMR spectroscopy. In this case, NMR spectra were acquired using 128 scans on a 600 MHz spectrometer with a BBFO probe operating at room temperature. By using nitrogen or helium cooled probes and increasing the number of scans for NMR measurements, the sensitivity could be improved. Furthermore, the sensitivity is also influenced by general factors such as available sample quantity, the sample matrix in general, the efficiency of the extraction method and the specific fractionation method and thus cannot be given in general terms. The tool was tested and validated using an artificial mixture of known compounds and was then successfully applied to a polar extract of a pine nut sample containing a large number of metabolites with high concentration differences. Fast and reliable identification of known and expected metabolites as well as the identification of rather unexpected metabolites such as *β*-d-glucopyranosyl-1-*N*-indole-3-acetyl-*N*-l-aspartic acid (**1**) and *N*^α^-(2-hydroxy-2-carboxymethylsuccinyl)-l-arginine (**2**) was shown. The presented method is not limited to the applicability of polar extracts, but can also be used in a wide range of metabolomics studies and natural product chemistry by minor adaptions such as the specific fractionation method.

## Materials and methods

### Reagents and chemicals

Deuteriumoxide (99.9%) and 3-(Trimethylsilyl)propionic-2,2,3,3-*d*_4_ acid sodium salt (TSP-*d*_4_, 99.0%) were purchased from Deutero (Kastellaun, Germany). Sodium azide (99.5%), potassium dihydrogen phosphate (≥ 99%), dipotassium hydrogen phosphate (≥ 98%) and formic acid (≥ 98%) were purchased from Sigma Aldrich (Merck KGaA, Darmstadt, Germany). Acetonitrile (≥ 99.9%) for Flash Chromatography was purchased from Fisher Chemical (Fisher Scientific GmbH, Schwerte, Germany). Ultrapure water for Flash Chromatography was purified by a Sartorius arium pro apparatur [Sartopore 0.2 µm, ultraviolet (UV)]. Acetonitrile (LiChrosolv^®^, Supelco^®^) and water (LiChrosolv^®^, Supelco^®^) for acquisition of MS spectra was purchased from Merck KGaA (Darmstadt, Germany). Methanol (≥ 99.8%) and 2-propanol (≥ 99.8%) were purchased from VWR International GmbH (Darmstadt, Germany). Sodium hydroxide (99.0%) was purchased from Grüssing GmbH (Filsum, Germany).

l-Isoleucine (> 99.5%), l-leucine (> 99.5%), l-phenylalanine (> 99.0%) and l-alanine (> 99.5%) were purchased from Fluka. Choline chloride (99%) was purchased from Fisher Scientific GmbH (Schwerte, Germany). Uridine (≥ 99%) was purchased from Carl Roth GmbH + Co. KG (Karlsruhe, Germany). Histamine (≥ 97%), *β*-alanine (99%) and creatinine (≥ 98%) were purchased from Sigma Aldrich (Merck KGaA, Darmstadt, Germany). d-(+)-Glucose monohydrate was purchased from Merck KGaA (Darmstadt, Germany).

### Sample preparation of the artificial mixture

For the artificial mixture containing ten compounds in total with different concentration courses, 0.1 M solutions of l-isoleucine, l-leucine, l-phenylalanine, l-alanine, choline chloride, uridine, histamine, *β*-alanine, creatinine and glucose in water (LC–MS grade) were prepared. According to Table S1, certain volumes of the solutions were combined and each sample was then filled up to 1 000 µL using water (LC–MS grade). From each sample, 50 µL were transferred into a new tube and diluted 500-fold for acquisition of mass spectra. The solvent of the remaining 950 µL of each sample was removed using a Speed Vacuum Concentrator (Savant SPD121P from Thermo Fisher Scientific, Schwerte, Germany). The residue was reconstituted in 700 µL phosphate buffer (200 mM, pH 7.0, 1 mM TSP-*d*_4_, 3 mM NaN_3_) and diluted tenfold. Then, 600 µL were transferred into NMR sample tube for acquisition of NMR spectra.

### Extraction of pine nut sample

The pine nut sample was purchased in a local grocery store in Hamburg (Germany). For sample preparation, 200 g of the pine nuts were shock-frozen with liquid nitrogen and ground with 300 g of dry ice using a Grindomix GM 300 knife mill equipped with a stainless-steel grinding container and a full metal knife (Retsch, Haan, Germany). The ground samples were freeze-dried for 48 h and stored at − 20 °C.

A suspension of 18.3 g pine nut lyophilizate and 180 mL of methanol was stirred for 2 h at room temperature. After removal of methanol under reduced pressure, 150 mL chloroform, 120 mL methanol and 180 mL bidistilled water were added and the suspension was stirred for 24 h. The suspension was centrifuged at 4 °C and 8000 rpm for 30 min (Centrifuge 5804R from Eppendorf™, Hamburg, Germany). The supernatant was collected and after evaporation of methanol, the extract was lyophilized. Then, the extract was reconstituted in 23 mL water and filtered through centrifugal filters with cutoff of 3 kDa (Amicon^®^ Ultra Centrifugal Filters) at 14 000 rcf and room temperature for 20 min (Centrifuge 5417R from Eppendorf™, Hamburg, Germany). Before use, the centrifugal filters were rinsed 20 times with 480 µL each of a 0.1 M sodium hydroxide solution and then once with 480 µL phosphate buffer. The filtrate of the pine nut extract was then lyophilized again and the dried extract was stored at − 20 °C.

### Flash chromatography

Fractionation of the pine nut extract was performed using a Büchi Sepacore^®^ Flash System with Control Unit C-620, UV Detector C-640 and Fraction Collector C-660. For separation, a CHROMABOND Flash DL 40 cartridge (Macherey–Nagel GmbH & Co. KG, Düren, Germany) packed with POLYGOPREP 60–30 NH2 LC packing material (Macherey–Nagel GmbH & Co. KG, Düren, Germany) was used. 514 mg of the dried pine nut extract was adsorbed onto 2.1 g of POLYGOPREOP 60–30 NH2 LC packing material. Bidistilled water was used as mobile phase A and acetonitrile as mobile phase B. A gradient under HILIC conditions was used, starting at 90% B and then decreasing linearly to 65% B in minutes 5 to 12. After holding 65% B for 3 min, proportion of B was further decreased to 0% within 12 min, then holding 0% B for 13 min. Total length of the flash chromatography method was 40 min. The flow rate was set to 20 mL/min. Fractions were collected with a volume of 10 mL each. A total of 80 fractions were collected.

### Sample preparation for NMR and MS measurements

Each fraction obtained after flash chromatography of the pine nut extract was lyophilized. The dried sample was then reconstituted in 1 000 µL water (LC–MS grade). For acquisition of mass spectra, 50 µL of the solution was transferred into a new tube and diluted 500-fold with water (LC–MS grade). For acquisition of NMR spectra, the remaining 950 µL of each fraction was lyophilized again and then reconstituted in 700 µL phosphate buffer (100 mM, pH 7.0, 1 mM TSP-*d*_4_, 3 mM NaN_3_) each. Then, 600 µL were transferred into NMR sample tube for measurement.

### Acquisition of NMR spectra

All NMR Spectra were acquired on a Bruker Avance III HD 600 MHz NMR spectrometer using TopSpin 3.6.2 (Bruker BioSpin GmbH, Rheinstetten, Germany) equipped with a 5 mm BBFO probe and operating at 600.13 MHz and 298 K. The HSQC and HMBC spectrum of (**2**) were acquired on a Bruker Avance NEO 600 MHz NMR spectrometer using TopSpin 4.1.3 (Bruker BioSpin GmbH, Rheinstetten, Germany) equipped with a 5 mm TCI Cryoprobe cooled with liquid nitrogen, operating at 600.25 MHz and 298 K.

The noesygppr1d pulse sequence was used for acquisition of all ^1^H NMR spectra applying water suppression. All spectra were recorded using a relaxation delay (D1) of 4 s, number of dummy scans (DS) of 4, number of data points (TD) of 65 536 and application of the digitization mode *baseopt*. For the NMR spectra of the artificial mixture 32 scans (NS) were recorded with a receiver gain (RG) of 64 and the transmitter frequency offset (O1) was set to 2824 Hz. The NMR spectra of the pine nut extract were recorded with NS of 128, O1 of 2820 Hz and RG of 32. Parameters of HSQC, HMBC and selective experiments are given in the respective figure captions in the Supporting Information.

### Acquisition of MS spectra

Mass spectra were acquired on a Bruker maXis ESI-Q-TOF mass spectrometer (maXis 4G, Bruker Daltonics, Bremen, Germany) coupled to Dionex Ultimate 3000 UPLC (Thermo Fisher Scientific, Schwerte, Germany). Measurements were performed using direct injection method (DI-MS) with water and acetonitrile, each containing 0.1% formic acid, as a mobile phase at a ratio of 50:50 and a flow rate of 0.2 mL/min with a total length of each measurement of 3 min. Injection volume was 10 µL for samples of the artificial mixture and 20 µL for samples of the pine nut extract. Mass Spectra were recorded in positive and negative ion mode with a mass range from *m/z* 50 to 2300. Mass spectra in positive (negative) ion mode were recorded using the following ESI source conditions: Capillary voltage: 4500 V (3000 V), End plate offset: − 500 V (− 500 V), drying gas flow: 8.0 L/min (8.0 L/min), drying gas temperature: 200 °C (200 °C), nebulizer gas: 5.0 bar (4.0 bar). Calibration of the mass spectrometer was performed before the start of the measurements using a sodium formate cluster solution as well as in the end of each individual DI-MS measurement of each sample by switching a valve and the syringe pump. The flow rate of the syringe pump was 0.1 mL/h.

### Data processing and analysis

#### NMR spectra

NMR spectra were processed using TopSpin 4.0.9 (Bruker BioSpin, Rheinstetten, Germany). The free induction decays (FIDs) were Fourier-transformed with an exponential function with line-broadening factor of 0.3 Hz. All ^1^H NMR spectra were calibrated to the TSP-*d*_4_ signal at 0.00 ppm and processed by automatic zero order phase correction (apk0) and automatic baseline correction (absn).

#### Mass spectra

The mass spectra were processed and analyzed using Compass Data Analysis 4.2 (Bruker Daltonics GmbH, Bremen, Germany). For all mass spectra acquired in positive and negative ionization mode, an average mass spectrum was calculated within the retention time range of minute 2.3 to 2.4 which only contains signals of the sodium formate cluster solution. This mass spectrum was then used for internal calibration of each sample automatically. Additionally, for all mass spectra, another average mass spectrum was calculated automatically from the total ion current (TIC) in the retention time range of minute 0.2 to 0.6 for each sample of each fraction. Text files consisting of *m/z* (two decimal places) vs. intensity for each sample were then exported for further analysis.

#### SCORE-metabolite-ID app

The correlation of NMR and MS data was performed using the self-developed SCORE-metabolite-ID app using the App Designer in MATLAB R2020b (TheMathworks, Inc., Natick, MA, USA). The MATLAB app is available upon request. NMR data of each fraction was imported using the script *rbnmr* by Nils Nyberg^34^. The NMR spectra were then calibrated to the TSP-*d*_4_ signal at 0.00 ppm and normalized to relative intensity of the TSP-*d*_4_ signal. If not otherwise stated, bucketing of the NMR spectra with a bucket size of 0.005 ppm was performed for data reduction. Mass spectra of each fraction were imported as text files of format *m/z* vs. intensity. Additionally, a mass spectrum of a blank measurement containing only water was also imported and intensities appearing in the blank spectrum were subtracted from each mass spectrum of each fraction.

### Supplementary Information

Supplementary Information.

## Data Availability

The data used to support the findings of this study are included within the article and supplementary materials.
