# Supplementary material for: Identification of metabolites from complex mixtures by 3D correlation of 1H NMR, MS and LC data using the SCORE-metabolite-ID approach
Source: Sci Rep. 2023 Sep 22;13:15834. doi: 10.1038/s41598-023-43056-3 (PMC10516956; doi:10.1038/s41598-023-43056-3)
Supplement: Supplementary file 1 — Supplementary Information. [file 41598_2023_43056_MOESM1_ESM.pdf]

Supporting Information for

Identification of metabolites from complex mixtures by 3D correlation of 1H NMR, MS and LC data using the SCORE-metabolite-ID approach

Stephanie Watermann[1], Marie-Christin Bode[1], Thomas Hackl[1,2,*]

[1] Institute of Organic Chemistry, University of Hamburg, Martin-Luther-King-Platz 6, 20146 Hamburg, Germany

[2] Hamburg School of Food Science – Institute of Food Chemistry, University of Hamburg, Grindelallee 117, 20146 Hamburg, Germany

[*] Corresponding author: Email: Thomas.hackl@chemie.uni-hamburg.de Tel.: +49-40 42838-2804

Table of Contents

–	**Table S1**	Preparation of the samples for the artificial mixture	S-III
–	**Table S2**	Correlation coefficients of EDCs of L-leucine and L-isoleucine in the artificial mixture	S-IV
–	**Table S3**	Comprehensive list of all identified metabolites in the polar extract of the pine nut sample including the respective correlation coefficients	S-V
–	**Table S4**	Semi-automatic detection of highly correlating EMCs to specific EDC of (**1**)	S-XII
–	**Table S5**	Correlation coefficients between all EDCs of (**1**)	S-XIII
–	**Table S6**	Correlation coefficients between EDCs and EMCs of (**1**)	S-XIV
–	**Table S7**	Semi-automatic detection of highly correlating EMCs to specific EDC of (**2**)	S-XV
–	**Table S8**	Correlation coefficients between EDCs and EMCs of (**2**)	S-XVI
–	**Figure S1**	DI-MS spectra of the artificial mixture	S-XVII
–	**Figure S2**	Selective TOCSY experiments of fraction 55 containing (**1**)	S-XVIII
–	**Figure S3**	HSQC and HMBC experiment of fraction 55 containing (**1**)	S-XIX
–	**Figure S4**	NMR spectra of fraction 67 containing (**2**) acquired in D_2O and in H_2O/D_2O (9:1)	S-XX
–	**Figure S5**	Selective TOCSY experiments of fraction 67 containing (**2**)	S-XXI
–	**Figure S6**	HSQC spectrum of fraction 68 containing (**2**)	S-XXII
–	**Figure S7**	HMBC spectrum of fraction 68 containing (**2**)	S-XXIII

Table S1: Amount of stock solution [µL] added to each sample, i.e. fraction, for the preparation of the artificial mixture. Stock solutions contain the pure compound each dissolved in water with a final concentration of 0.1 M. Ile: L-Isoleucine, Leu: L-Leucine, Cho.: Choline, Urid.: Uridine, Creat.: Creatinine, Ala: L-Alanine, Phe: L-Phenylalanine, Glc: D-Glucose, *β*-Ala: *β*-Alanine, Hist.: Histamine.

Fract.	Ile	Leu	Cho.	Urid.	Creat.	Ala	Phe	Glc	*β*-Ala	Hist.
**1**	10	0	0	0	0	0	0	0	0	0
**2**	30	10	0	0	0	0	0	0	0	0
**3**	80	30	0	0	0	0	0	0	0	0
**4**	130	80	0	20	0	0	0	0	0	0
**5**	130	130	0	40	0	0	0	0	0	0
**6**	80	130	0	80	0	0	0	0	0	0
**7**	30	80	5	120	0	0	0	0	0	0
**8**	10	30	10	160	0	0	0	0	0	0
**9**	0	10	20	200	0	0	0	0	0	0
**10**	0	0	25	220	0	0	0	0	0	0
**11**	0	0	20	210	0	0	0	0	0	0
**12**	0	0	10	160	20	20	0	0	0	0
**13**	0	0	5	115	80	80	0	0	0	0
**14**	0	0	0	70	180	200	10	0	0	0
**15**	0	0	0	5	160	80	50	0	0	0
**16**	0	0	0	0	130	20	130	0	0	0
**17**	0	0	0	0	100	0	180	10	0	0
**18**	0	0	0	0	70	0	130	100	0	0
**19**	0	0	0	0	20	0	50	10	0	0
**20**	0	0	0	0	0	0	10	0	20	5
**21**	0	0	0	0	0	0	0	0	70	50
**22**	0	0	0	0	0	0	0	0	95	150
**23**	0	0	0	0	0	0	0	0	100	350
**24**	0	0	0	0	0	0	0	0	95	150
**25**	0	0	0	0	0	0	0	0	70	50
**26**	0	0	0	0	0	0	0	0	20	5

Table S2: Pearson Correlation Coefficients calculated between NMR signals of L-Leucine and L-Isoleucine in the artificial mixture over all 26 fractions. Coefficients > 0.95 are highlighted green.

		L-Leucine			L-Isoleucine				
	**[ppm]**	**0.956**	**1.721**	**3.739**	**0.944**	**1.021**	**1.271**	**1.987**	**3.675**
**L-Leucine**	**0.956**		0.9770	0.9725	0.9541	0.9393	0.9383	0.9427	0.9419
	**1.721**	0.9770		0.9954	0.8689	0.8450	0.8444	0.8506	0.8526
	**3.739**	0.9725	0.9954		0.8657	0.8400	0.8377	0.8456	0.8465
**L-Iso-leucine**	**0.944**	0.9541	0.8689	0.8657		0.9968	0.9953	0.9973	0.9913
	**1.021**	0.9393	0.8450	0.8400	0.9968		0.9994	0.9998	0.9970
	**1.271**	0.9383	0.8444	0.8377	0.9953	0.9994		0.9993	0.9974
	**1.987**	0.9427	0.8506	0.8456	0.9973	0.9998	0.9993		0.9974
	**3.675**	0.9419	0.8526	0.8465	0.9913	0.9970	0.9974	0.9974	

List of further identified metabolites

In total, 40 metabolites were identified in the polar extract of pine nuts. The following table S3 shows the calculated correlation coefficients of 38 of the metabolites. Correlation coefficients of *β*-D-glucopyranosyl-1-*N*-indole-3-acetyl-*N*-L-aspartic acid (**1**) and N^{α}-(2-hydroxy-2-carboxymethylsuccinyl)-L-arginine (**2**) are listed in tables S4-S8. In most cases, a bucket size of 0.005 ppm was used for bucketing. A larger bucket size usually was used to compensate for pH shifts of signals over fractions, e.g., as observed for citrate. A different bucket size is indicated in the table entry. Depending on the complexity of the spectra and complexity of the metabolites, the metabolite identification is not only based on the correlation coefficients, but also, for example, on splitting patterns and coupling constants of the NMR signals, further information from selective or 2D NMR experiments or LC-MS runs. The semi-automatic correlation of NMR and MS signals often yields more high correlation factors than can be reasonably assigned. Meaningful molecular ion adducts, and fragment ions tend to be obtained for high correlations with high signal intensities. Therefore, such signals are highlighted in the app. In all of the following tables, correlation coefficients > 0.95 are also highlighted green. Lower correlation coefficients result mainly from signal overlap of other metabolites in the NMR spectra of successive fractions or from low-intensity mass signals in the MS spectra. For all metabolites shown, the interpretation of the data is not based solely on the correlation factors but requires an in-depth interpretation of the data.

Table S3: List of further identified metabolites in the polar extract of pine nuts. The left column lists the metabolites together with indication of the exact mass as well as the range of fractions over which the correlation coefficients were calculated. The right column each contains tables of correlation coefficients between specific EDCs and EMCs or specific EDCs with each other. Correlation coefficients > 0.95 are highlighted green.

Metabolite | **Correlation Coefficients**

AMINO ACIDS

L-Alanine
89.0477 Da
F1-80

ESI-(+)	90.05 ([M+H]$^+$)
**1.489 ppm**	0.9618

L-Arginine
174.1117 Da
F35-80

ESI-(+)	175.12 ([M+H]$^+$)	157.11 ([M-NH$_3$+H]$^+$)	60.05 (protonated guanidine)
**3.246 ppm**	0.9789	0.9789	0.9903
**1.931 ppm**	0.9817	0.9750	0.9849
**1.654 ppm**	0.9724	0.9688	0.9823

L-Asparagine
132.0535 Da
F40-50

ESI-(+)	133.06 ([M+H]$^+$)	87.05 ([M-H$_2$O-CO+H]$^+$)
**2.940 ppm**	0.9888	0.9864
**2.875 ppm**	0.9888	0.9862

L-Aspartic acid
133.0375 Da
F46-66

ESI-(+)	134.04 ([M+H]$^+$)	116.03 ([M-H$_2$O+H]$^+$)	88.04 ([M-H$_2$O-CO+H]$^+$)	70.03 ([M-2H$_2$O-CO+H]$^+$)
**2.799 ppm**	0.9948	0.9719	0.9720	0.9649
**2.704 ppm**	0.9948	0.9721	0.9738	0.9680

L-Glutamic acid
147.0532 Da
F46-80

ESI-(+)	148.06 ([M+H]$^+$)	102.05 ([M-H$_2$O-CO+H]$^+$)	84.04 ([M-2H$_2$O-CO+H]$^+$)
**2.354 ppm**	0.9844	0.9812	0.9808
**2.119 ppm**	0.9865	0.9829	0.9830
**2.065 ppm**	0.9920	0.9882	0.9883

Metabolite	Correlation Coefficients

L-Glutamine

146.0691 Da
F41-47

ESI-(+)	147.08 ([M+H]$^+$)	101.07 ([M-H_2O-CO+H]$^+$)
**2.464 ppm**	0.9411	0.9583
**2.150 ppm**	0.8691	0.8939

L-Histidine

155.0695 Da
F40-50

ESI-(+)	156.08 ([M+H]$^+$)	178.06 ([M+Na]$^+$)	110.07 ([M-H_2O-CO+H]$^+$)
**7.769 ppm**	0.9325	0.9704	0.9432
**7.122 ppm**	0.9920	0.9626	0.9872

L-Isoleucine

131.0946 Da
F30-37

EDC [ppm]	1.4768	1.9867	1.0097	0.9437
**1.4768**	-	0.9977	0.9942	0.9836
**1.9867**	0.9977	-	0.9858	0.9721
**1.0097**	0.9942	0.9858	-	0.9956
**0.9437**	0.9836	0.9721	0.9956	-

L-Leucine

131.0946 Da
F30-37

EDC [ppm]	1.7068	0.9662
**1.7068**	-	0.9967
**0.9662**	0.9967	-

L-Lysine

146.1055 Da
F48-62

ESI-(+)	147.11 ([M+H]$^+$)	130.09 ([M-NH_3+H]$^+$)	84.08 ([M-NH_3-H_2O-CO+H]$^+$)
**3.041 ppm**	0.9554	0.9605	0.9711
**1.734 ppm**	0.9138	0.9160	0.9336
**1.469 ppm**	0.8452	0.8574	0.8720

L-Methionine

149.0510 Da
F34-41

ESI-(+)	150.06 ([M+H]$^+$)
**2.646 ppm**	0.9938
**2.139 ppm**	0.9472

L-Phenylalanine

165.0790 Da
F1-41

ESI-(+)	166.09 ([M+H]$^+$)	120.08 ([M-H_2O-CO+H]$^+$)
**7.435 ppm**	0.9787	0.9777
**7.380 ppm**	0.9945	0.9930
**7.340 ppm**	0.9962	0.9974

L-Proline

115.0633 Da
F37-43

ESI-(+)	116.07 ([M+H]$^+$)	70.06 ([M-H_2O-CO+H]$^+$)
**4.136 ppm**	0.9946	0.9867
**3.351 ppm**	0.9394	0.9537
**3.413 ppm**	0.9490	0.9258
**2.336 ppm**	0.9927	0.9832
**2.071 ppm**	0.9893	0.9909
**1.991 ppm**	0.9834	0.9689

L-Threonine

119.0582 Da
F40-48

ESI-(+)	74.06 ([M-H_2O-CO+H]$^+$)
**1.340 ppm**	0.9666

L-Tryptophane

204.0899 Da
F1-39

ESI-(+)	205.10 ([M+H]$^+$)	227.08 ([M+Na]$^+$)	188.07 ([M-NH_3+H]$^+$)	146.07
**7.735 ppm**	0.9907	0.9822	0.9975	0.9966
**7.540 ppm**	0.9915	0.9901	0.9960	0.9957
**7.290 ppm**	0.9920	0.9840	0.9980	0.9972
**7.205 ppm**	0.9923	0.9821	0.9981	0.9969

Metabolite	Correlation Coefficients

L-Tyrosine

181.0739 Da
F36-80

ESI-(+)	182.08 ($[M+H]^+$)	165.05 ($[M-NH_3+H]^+$)	136.08 ($[M-H_2O-CO+H]^+$)	119.05 ($[M-H_2O-CO-NH_3+H]^+$)
**7.190 ppm**	0.9814	0.9951	0.9743	0.9956
**6.910 ppm**	0.9825	0.9989	0.9786	0.9956

L-Valine

117.0790 Da
F36-41

ESI-(+)	118.09 ($[M+H]^+$)	72.08 ($[M-H_2O-CO+H]^+$)
**2.280 ppm**	0.9826	0.8073
**1.039 ppm**	0.9681	0.9596
**1.000 ppm**	0.9838	0.9414

Glutathione (oxidized)

612.1520 Da
F65-72

ESI-(+)	613.15 ($[M+H]^+$)	307.08 ($[M+2H]^{2+}$)
**3.770 ppm**	0.8139	0.8440
**2.976 ppm**	0.9321	0.9569
**2.175 ppm**	0.9007	0.9283

ESI-(-)	611.15 ($[M-H]^-$)
**3.770 ppm**	0.8854
**2.976 ppm**	0.9807
**2.175 ppm**	0.9549

CARBOHYDRATES AND ALDITOLS

Glycerol

92.0473 Da
F1-30

EDC [ppm]	3.779	3.665	3.641	3.546
**3.779**	-	0.9125	0.9179	0.9129
**3.665**	0.9125	-	0.9985	0.9995
**3.641**	0.9179	0.9985	-	0.9987
**3.546**	0.9129	0.9995	0.9984	-

D-Pinitol

194.0790 Da
F1-37

ESI-(+)	195.09 ($[M+H]^+$)	217.07 ($[M+Na]^+$)	109.03 ($[M+H+Na]^{2+}$)	411.15 ($[2M+Na]^+$)
**4.013 ppm**	0.9859	0.9861	0.9865	0.9874
**3.809 ppm**	0.9660	0.9733	0.9831	0.9648
**3.772 ppm**	0.9426	0.9557	0.9739	0.9388
**3.656 ppm**	0.9804	0.9861	0.9894	0.9811
**3.599 ppm**	0.9757	0.9845	0.9887	0.9758
**3.349 ppm**	0.9810	0.9828	0.9851	0.9824

ESI-(-)	193.07 ($[M-H]^-$)	239.08 ($[M+FA-H]^-$)	222.10
**4.013 ppm**	0.9933	0.9854	0.9924
**3.809 ppm**	0.9757	0.9696	0.9796
**3.772 ppm**	0.9552	0.9509	0.9568
**3.656 ppm**	0.9916	0.9847	0.9923
**3.599 ppm**	0.9895	0.9839	0.9864
**3.349 ppm**	0.9898	0.9815	0.9893

Metabolite	Correlation Coefficients

Raffinose

504.1690 Da
F1-80

ESI-(+)	527.16 ([M+Na]$^+$)	543.13 ([M+K]$^+$)
**5.435 ppm**	0.9807	0.9693
**4.996 ppm**	0.9749	0.9653
**4.236 ppm**	0.9797	0.9771
**4.086 ppm**	0.9503	0.9367

ESI-(-)	503.17 ([M-H]$^-$)	549.17 ([M+FA-H]$^-$)	617.16 ([M+TFA-H]$^-$)
**5.435 ppm**	0.9802	0.9837	0.9942
**4.996 ppm**	0.9703	0.9746	0.9839
**4.236 ppm**	0.9755	0.9761	0.9593
**4.086 ppm**	0.9495	0.9548	0.9807

Sucrose*

342.1162 Da
F1-80

ESI-(+)	365.11 ([M+Na]$^+$)	707.23 ([2M+Na]$^{2+}$)
**5.412 ppm**	0.9531	0.9929
**4.226 ppm**	0.9524	0.9869
**4.051 ppm**	0.8678	0.9294
**3.896 ppm**	0.9406	0.9509
**3.857 ppm**	0.9688	0.9673
**3.821 ppm**	0.9630	0.9928
**3.767 ppm**	0.9629	0.9510
**3.676 ppm**	0.8638	0.9251
**3.567 ppm**	0.9479	0.9774
**3.473 ppm**	0.8919	0.9525

ESI-(-)	341.10 ([M-H]$^-$)	387.11 ([M+FA-H]$^-$)	683.21 ([2M-H]$^{2-}$)	729.21 ([2M+FA-H]$^{2-}$)
**5.412 ppm**	0.9812	0.9833	0.9967	0.9974
**4.226 ppm**	0.9781	0.9780	0.9862	0.9874
**4.051 ppm**	0.8992	0.9013	0.9681	0.9644
**3.896 ppm**	0.9488	0.9448	0.9486	0.9495
**3.857 ppm**	0.9840	0.9808	0.9430	0.9477
**3.821 ppm**	0.9846	0.9858	0.9935	0.9945
**3.767 ppm**	0.9738	0.9691	0.9178	0.9236
**3.676 ppm**	0.8883	0.8911	0.9614	0.9571
**3.567 ppm**	0.9691	0.9679	0.9800	0.9807
**3.473 ppm**	0.9220	0.9257	0.9832	0.9802

*MS Spectra were acquired in 3000-fold dilution.

ORGANIC ACIDS

Citric acid*

192.0270 Da
F60-80

ESI-(-)	191.02 ([M-H]$^-$)	213.00 ([M+Na-2H]$^-$)
**2.70 ppm**	0.7956	0.7244
**2.57 ppm**	0.9015	0.8798
**2.54 ppm**	0.8805	0.8563

*Bucketing of NMR spectra was performed with a larger bucket size of 0.03 ppm because of strong signal shifts.

Metabolite	Correlation Coefficients

Malic acid

134.0215 Da
F50-67

ESI-(+)	157.01 ([M+Na]$^+$)	172.98 ([M+K]$^+$)	178.99 ([M+2Na-H]$^+$)
**4.289 ppm**	0.9837	0.9800	0.9634
**2.659 ppm**	0.9834	0.9765	0.9579
**2.384 ppm**	0.9780	0.9633	0.9447

ESI-(-)	133.01 ([M-H]$^-$)	155.00 ([M+Na-2H]$^-$)	115.00 ([M-H_2O-H]$^-$)
**4.289 ppm**	0.9817	0.9850	0.9794
**2.659 ppm**	0.9787	0.9878	0.9829
**2.384 ppm**	0.9714	0.9755	0.9868

Nicotinic acid

123.0320 Da
F39-47

ESI-(+)	124.04 ([M+H]$^+$)
**8.942 ppm**	0.9874
**8.612 ppm**	0.9957
**8.250 ppm**	0.9932
**7.542 ppm**	0.9584

ESI-(-)	122.03 ([M-H]$^-$)
**8.942 ppm**	0.9820
**8.612 ppm**	0.9639
**8.250 ppm**	0.9552
**7.542 ppm**	0.8968

BETAINES

Betaine

117.0790 Da
F21-34

ESI-(+)	118.09 ([M+H]$^+$)
**3.269 ppm**	0.9503

Choline

104.1070 Da
F1-36

ESI-(+)	104.11 ([M$^+$])
**4.069 ppm**	0.9983
**3.531 ppm**	0.9695
**3.206 ppm**	0.9894

Ergothioneine

229.0885 Da
F1-80

ESI-(+)	230.10 ([M+H]$^+$)	252.08 ([M+Na]$^+$)	268.05 ([M+K]$^+$)	274.06 ([M+2Na-H]$^+$)
**6.804 ppm**	0.9989	0.9866	0.9541	0.9600
**3.276 ppm**	0.9958	0.9834	0.9606	0.9527

ESI-(-)	228.08 ([M-H]$^-$)
**6.804 ppm**	0.9942
**3.276 ppm**	0.9856

Phosphoryl-choline

183.0660 Da
F49-80

ESI-(+)	184.07 ([M+H]$^+$)	206.05 ([M+Na]$^+$)	228.04 ([M+2Na-H]$^+$)
**4.176 ppm**	0.9792	0.9850	0.9729
**3.601 ppm**	0.9754	0.9818	0.9710
**3.226 ppm**	0.9874	0.9944	0.9914

Trigonelline

137.0477 Da
F27-38

ESI-(+)	138.06 ([M+H]$^+$)
**9.13 ppm**	0.9810
**8.84 ppm**	0.9851
**8.08 ppm**	0.9817
**4.44 ppm**	0.9680

Metabolite	Correlation Coefficients
	**NUCLEOSIDES, NUCLEOTIDES AND NUCLEOBASES**

Adenine*

135.0545 Da
F1-37

ESI-(+)	136.06 ([M+H]+)	158.04 ([M+Na]+)
**8.254 ppm**	0.9506	0.9751
**8.201 ppm**	0.9509	0.9805

ESI-(-)	134.05 ([M-H]-)
**8.254 ppm**	0.9905
**8.201 ppm**	0.9928

*Bucketing of NMR spectra was performed with a larger bucket size of 0.01 ppm because of signal shifts.

Adenosine

267.0968 Da
F1-28

ESI-(+)	268.10 ([M+H]+)	290.09 ([M+Na]+)
**8.347 ppm**	0.9943	0.9992
**8.267 ppm**	0.9750	0.9915
**6.077 ppm**	0.9791	0.9949
**4.300 ppm**	0.9005	0.9367

ESI-(-)	266.09 ([M-H]-)	302.07 ([M+Cl]-)	312.10 ([M+FA-H]-)
**8.347 ppm**	0.9930	0.9978	0.9931
**8.267 ppm**	0.9980	0.9767	0.9990
**6.077 ppm**	0.9980	0.9842	0.9978
**4.300 ppm**	0.9583	0.9081	0.9585

Adeonsine 5'-monophosphate

347.0631 Da
F65-75

ESI-(+)	348.07 ([M+H]+)	370.05 ([M+Na]+)	392.03 ([M+2Na-H]+)	136.06 (Adenine fragment)
**8.602 ppm**	0.9738	0.9499	0.9139	0.9742
**8.272 ppm**	0.9856	0.9667	0.9298	0.9909
**6.152 ppm**	0.9822	0.9628	0.9254	0.9886
**4.511 ppm**	0.9841	0.9674	0.9340	0.9888
**4.371 ppm**	0.9734	0.9498	0.9122	0.9779
**4.031 ppm**	0.9740	0.9577	0.9267	0.9794

ESI-(-)	346.06 ([M-H]-)
**8.602 ppm**	0.9764
**8.272 ppm**	0.9906
**6.152 ppm**	0.9903
**4.511 ppm**	0.9919
**4.371 ppm**	0.9812
**4.031 ppm**	0.9867

Citicoline

488.1073 Da
F46-54

ESI-(+)	489.11 ([M+H]+)	511.10 ([M+Na]+)	533.08 ([M+2Na-H]+)
**7.942 ppm**	0.8694	0.8828	0.8946
**6.137 ppm**	0.9102	0.9088	0.9120
**5.997 ppm**	0.9544	0.9754	0.9716

Metabolite	Correlation Coefficients

Guanosine

283.0917 Da
F32-42

ESI-(+)	284.10 ([M+H]$^{+}$)	306.08 ([M+Na]$^{+}$)	152.06 (Guanine fragment)
**8.005 ppm**	0.9518	0.9783	0.9920

ESI-(-)	282.09 ([M-H]$^{-}$)
**8.005 ppm**	0.9822

Nicotinamide adenine dinucleotide (NAD)

663.1091 Da
F46-65

ESI-(+)	664.12 ([M+H]$^{+}$)	332.56 ([M+2H]$^{2+}$)
**9.343 ppm**	0.9380	0.9848
**9.142 ppm**	0.9451	0.9878
**8.847 ppm**	0.9256	0.9396
**8.432 ppm**	0.9501	0.9908
**8.196 ppm**	0.9246	0.9680
**8.179 ppm**	0.9381	0.9849
**6.122 ppm**	0.9541	0.9093
**6.007 ppm**	0.9587	0.9296

Uridine

244.0695 Da
F28-34

ESI-(+)	267.06 ([M+Na]$^{+}$)	289.04 ([M+2Na-H]$^{+}$)
**7.887 ppm**	0.9916	0.9982
**5.916 ppm**	0.9918	0.9968
**4.360 ppm**	0.9382	0.9647

ESI-(-)	243.07 ([M-H]$^{-}$)	279.04 ([M+Cl]$^{-}$)	289.07 ([M+FA-H]$^{-}$)
**7.887 ppm**	0.9969	0.9909	0.9843
**5.916 ppm**	0.9948	0.9903	0.9854
**4.360 ppm**	0.9653	0.9717	0.9613

Uridine 5'-monophosphate

324.0359 Da
F64-77

ESI-(+)	325.04 ([M+H]$^{+}$)	347.02 ([M+Na]$^{+}$)
**8.102 ppm**	0.9063	0.8965
**5.997 ppm**	0.9279	0.8909
**5.982 ppm**	0.9246	0.8380
**4.423 ppm**	0.9315	0.9003

ESI-(-)	611.15 ([M-H]$^{-}$)
**8.102 ppm**	0.9311
**5.997 ppm**	0.9377
**5.982 ppm**	0.9027
**4.423 ppm**	0.9487

Table S4: Eleven most intense EMC signals in positive ionization mode that show correlation coefficient > 0.96 to EDC at 7.485 ppm of (**1**) in fractions 51-65 of the polar pine nut extract including the peak assignment.

**MS signal [*m/z*]**	**Pearson correlation coefficient**	**Maximum intensity of MS signal**	**Peak assignment**
291.10	0.9734	156698	fragment I
475.13	0.9846	106347	$[M+Na]^+$
247.04	0.9681	77998	
292.10	0.9663	35366	fragment I ^{13}C-isotope
246.05	0.9681	31840	
491.10	0.9701	26588	$[M+K]^+$
157.01	0.9777	18795	
200.97	0.9856	17856	
453.15	0.9609	16976	$[M+H]^+$
333.11	0.9647	14752	fragment II
497.11	0.9895	14182	$[M+2Na-H]^+$

Table S5: Pearson Correlation Coefficients of several EDCs of (**1**) in fractions 50-65 of the polar pine nut extract. Coefficients > 0.96 are highlighted green.

[ppm]	**7.672**	**7.621**	**7.485**	**7.355**	**7.265**	**5.624**	**4.435**	**4.099**	**2.636**	**2.509**
**7.672**		0.9982	0.9985	0.9928	0.9997	0.9805	0.9946	0.9933	0.9955	0.9907
**7.621**	0.9982		0.9995	0.9969	0.9972	0.9838	0.9960	0.9929	0.9964	0.9875
**7.485**	0.9985	0.9995		0.9959	0.9976	0.9790	0.9936	0.9917	0.9940	0.9866
**7.355**	0.9928	0.9969	0.9959		0.9921	0.9789	0.9949	0.9915	0.9941	0.9863
**7.265**	0.9997	0.9972	0.9976	0.9921		0.9785	0.9947	0.9946	0.9955	0.9928
**5.624**	0.9805	0.9838	0.9790	0.9789	0.9785		0.9878	0.9740	0.9887	0.9683
**4.435**	0.9946	0.9960	0.9936	0.9949	0.9947	0.9878		0.9957	0.9998	0.9941
**4.099**	0.9933	0.9929	0.9917	0.9915	0.9946	0.9740	0.9957		0.9959	0.9960
**2.636**	0.9955	0.9964	0.9940	0.9941	0.9955	0.9887	0.9998	0.9959		0.9936
**2.509**	0.9907	0.9875	0.9866	0.9863	0.9928	0.9683	0.9941	0.9960	0.9936	

Table S6: Pearson correlation coefficients of EDCs and EMCs in positive ionization mode of compound (**1**) calculated in fractions 51-65. Coefficients > 0.95 are highlighted green.

		**MS signals [*m/z*]**				
		491.10	475.13	453.15	333.11	291.10
**NMR signals [ppm]**	7.672	0.9802	0.9917	0.9730	0.9762	0.9828
	7.621	0.9677	0.9829	0.9581	0.9638	0.9714
	7.485	0.9701	0.9846	0.9609	0.9647	0.9734
	7.355	0.9532	0.9720	0.9447	0.9563	0.9583
	7.265	0.9817	0.9925	0.9761	0.9789	0.9836
	5.624	0.9477	0.9641	0.9321	0.9474	0.9574
	4.435	0.9631	0.9789	0.9567	0.9694	0.9675
	4.099	0.9664	0.9793	0.9643	0.9699	0.9662
	2.636	0.9662	0.9810	0.9595	0.9703	0.9700
	2.509	0.9705	0.9821	0.9719	0.9822	0.9710

Table S7: Five most intense EMC signals in positive ionization mode that show correlation coefficient > 0.96 to EDC at 2.456 ppm of (**2**) in fractions 60-73 of the polar pine nut extract including the peak assignment.

**MS signal [*m/z*]**	**Pearson correlation coefficient**	**Maximum intensity of MS signal**	**Peak assignment**
349.13	0.9933	173091	$[M+H]^+$
350.14	0.9936	26363	$[M+H]^+$ isotope
175.12	0.9737	22033	fragment
351.14	0.9629	6195	$[M+H]^+$ isotope
393.10	0.9650	3576	$[M+2Na-H]^+$

Table S8: Pearson correlation coefficients of EDCs and EMCs in positive and negative ionization mode of compound (**2**) calculated in fractions 60-73. Coefficients > 0.95 are highlighted green.

		**MS signals [*m/z*]**			
		349.13 ($[M+H]^+$)	175.12 ($[M+2Na-H]^+$)	347.12 ($[M-H]^-$)	329.11 ($[M-H_2O-H]^-$)
**NMR signals [ppm]**	4.241	0.9685	0.9643	0.9719	0.9674
	3.196	0.9936	0.9798	0.9946	0.9755
	2.651	0.9713	0.9176	0.9689	0.9662
	2.456	0.9933	0.9737	0.9938	0.9757
	1.876	0.9559	0.9709	0.9602	0.9438
	1.636	0.9700	0.9702	0.9724	0.9517
	1.566	0.9611	0.9642	0.9642	0.9438

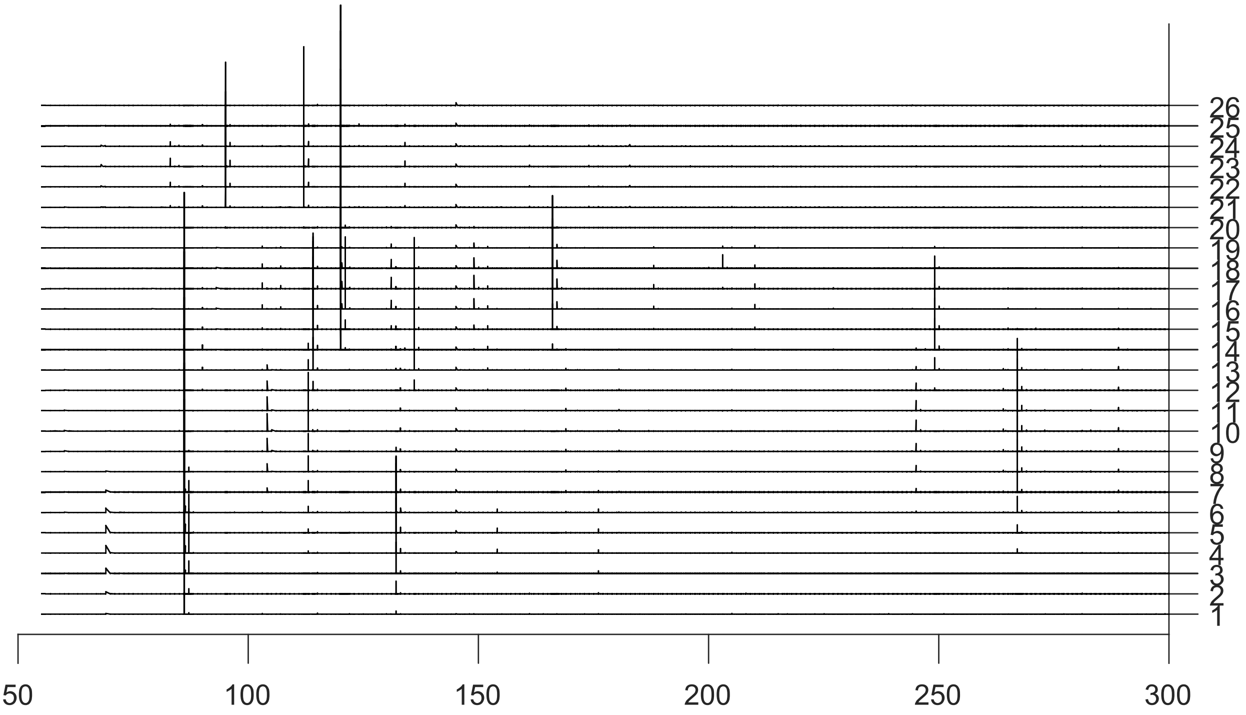

Figure S1: DI-MS spectra in positive ionization mode of the artificial mixture.

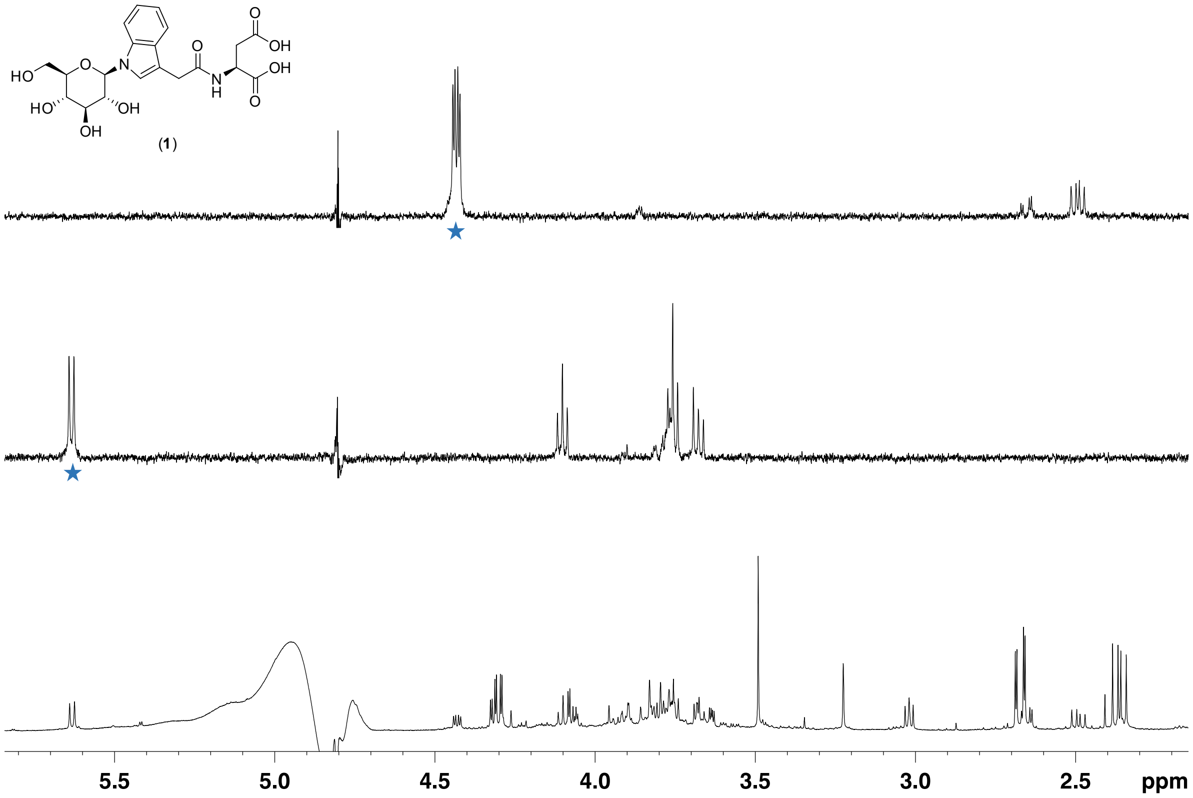

Figure S2: Selective TOCSY experiments of fraction 55 of the polar pine nut extract. The NMR spectrum of the whole fraction 55 is shown in the lower part. The irradiated frequency for selective excitation in the two upper spectra is marked by an asterisk. The spectrum in the middle shows signals of the spin system of D-glucose. The upper spectrum shows signal of an ABX spin system, which could be identified as L-aspartic acid. The selective TOCSY experiments were acquired using the seldigpzs pulse sequence with 512 scans, 4 dummy scans and 32 768 data points.

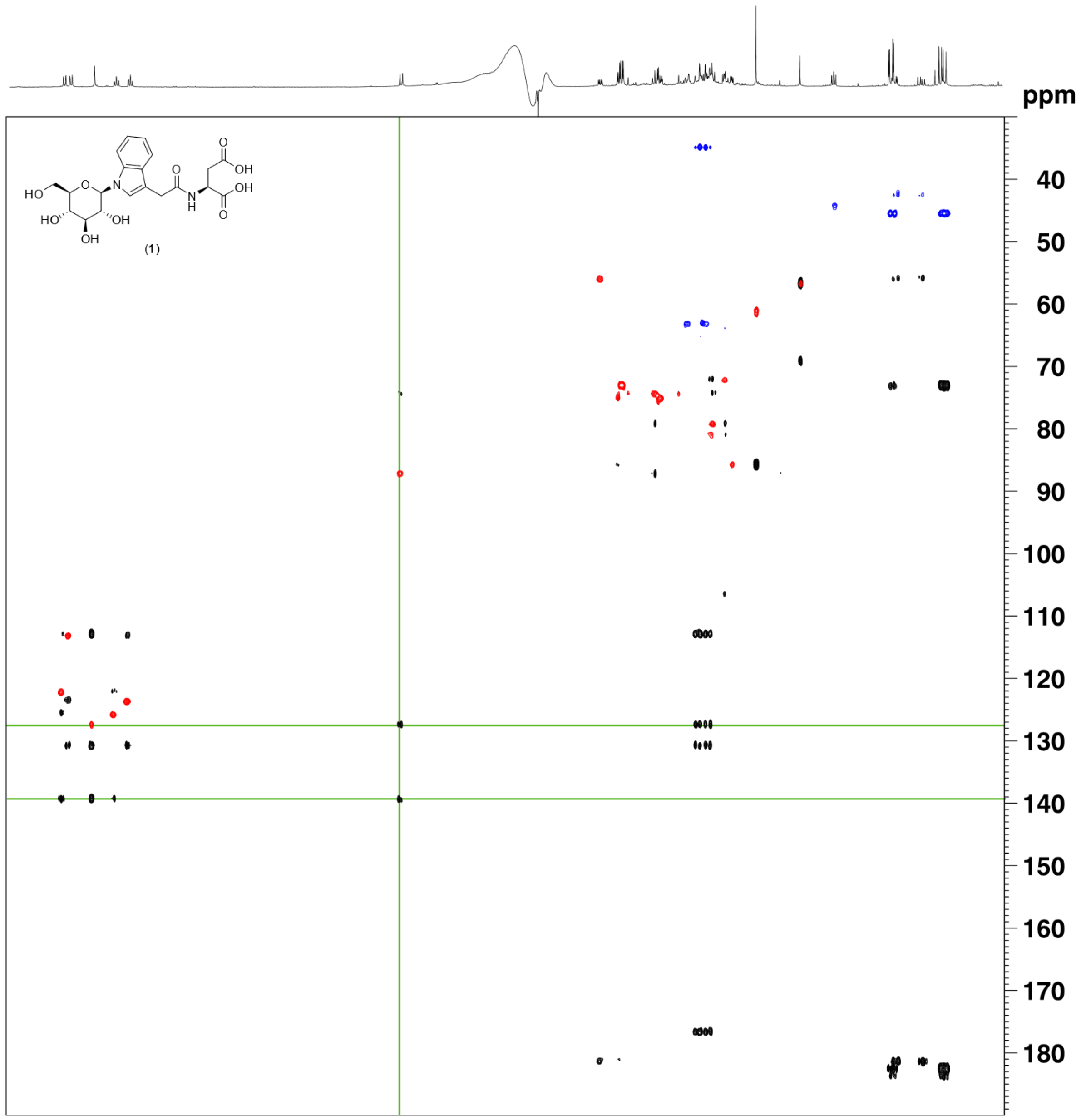

Figure S3: Part of the 2D HSQC spectrum (red and blue peaks) and 2D HMBC spectrum (black peaks) of fraction 55 of polar pine nut extract containing IAA-Asp-*N*-Glc (**1**). The correlation peaks between H-1' (5.63 ppm, anomeric proton of glucose) to C-2 (127.2 ppm) and C-7a (139.4 ppm), which confirm the glucose-*N*-indole bond, are highlighted green. For the acquisition of the HSQC spectrum the hsqcedetgpsp.3 pulse sequence was used. The spectrum was acquired with 2048 data points in F2 and 256 increments. 32 dummy scans and 128 scans were acquired. The HMBC spectrum was acquired using the hmbcetgpl3nd pulse sequence with 4096 data points in F2 and 256 increments. 16 dummy scans and 256 scans were acquired.

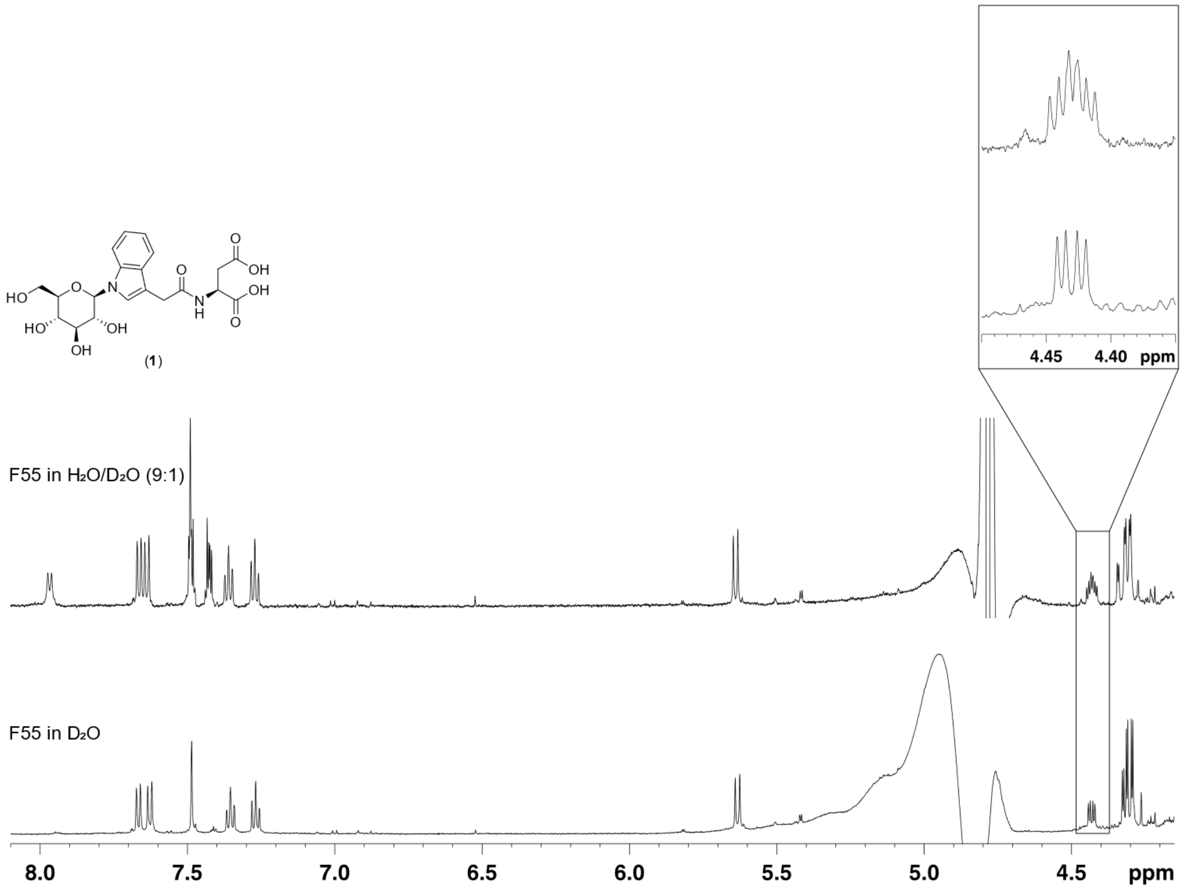

Figure S4: ^{1}H NMR spectra of fraction 55 of polar pine nut extract containing IAA-Asp-*N*-Glc (**1**) in phosphate buffer in D_2O (lower spectrum) and in H_2O/D_2O (9:1) (upper spectrum). An additional doublet at 7.97 ppm appears in NMR spectrum acquired in H_2O/D_2O. Furthermore, multiplicity of signal at 4.43 ppm changes from dd in D_2O to ddd in H_2O/D_2O because of the additional coupling to amide proton at 7.97 ppm.

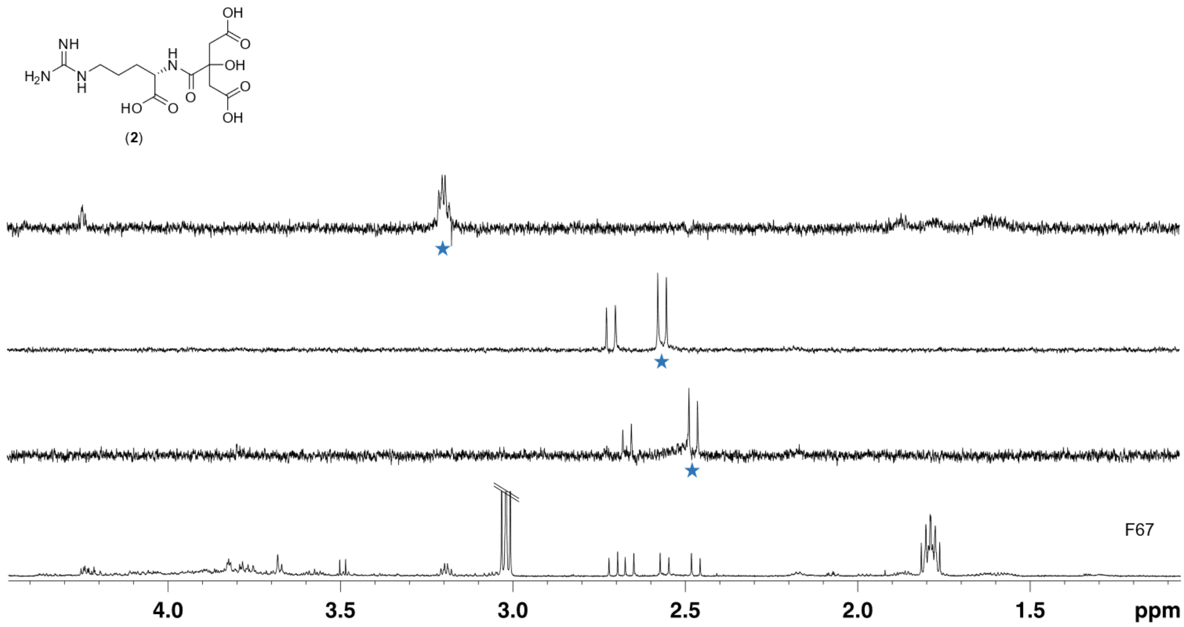

Figure S5: Selective TOCSY experiments of fraction 67 of the polar pine nut extract. The lowest spectrum shows the ^{1}H NMR spectrum of the whole fraction 67. Irradiated frequencies for selective excitation of the TOCSY experiments (upper three spectra) are marked by an asterisk. The three spin systems of the three selective TOCSY experiments show high correlation coefficients over a series of fractions. The compound was identified as condensation product of citric acid and L-arginine, N^{α}-(2-hydroxy-2-carboxymethylsuccinyl)-L-arginine (**2**). The selective TOCSY spectra were acquired using the seldigpzs pulse sequence. For all three spectra, 4 dummy scans, 512 scans and 32 768 data points were acquired.

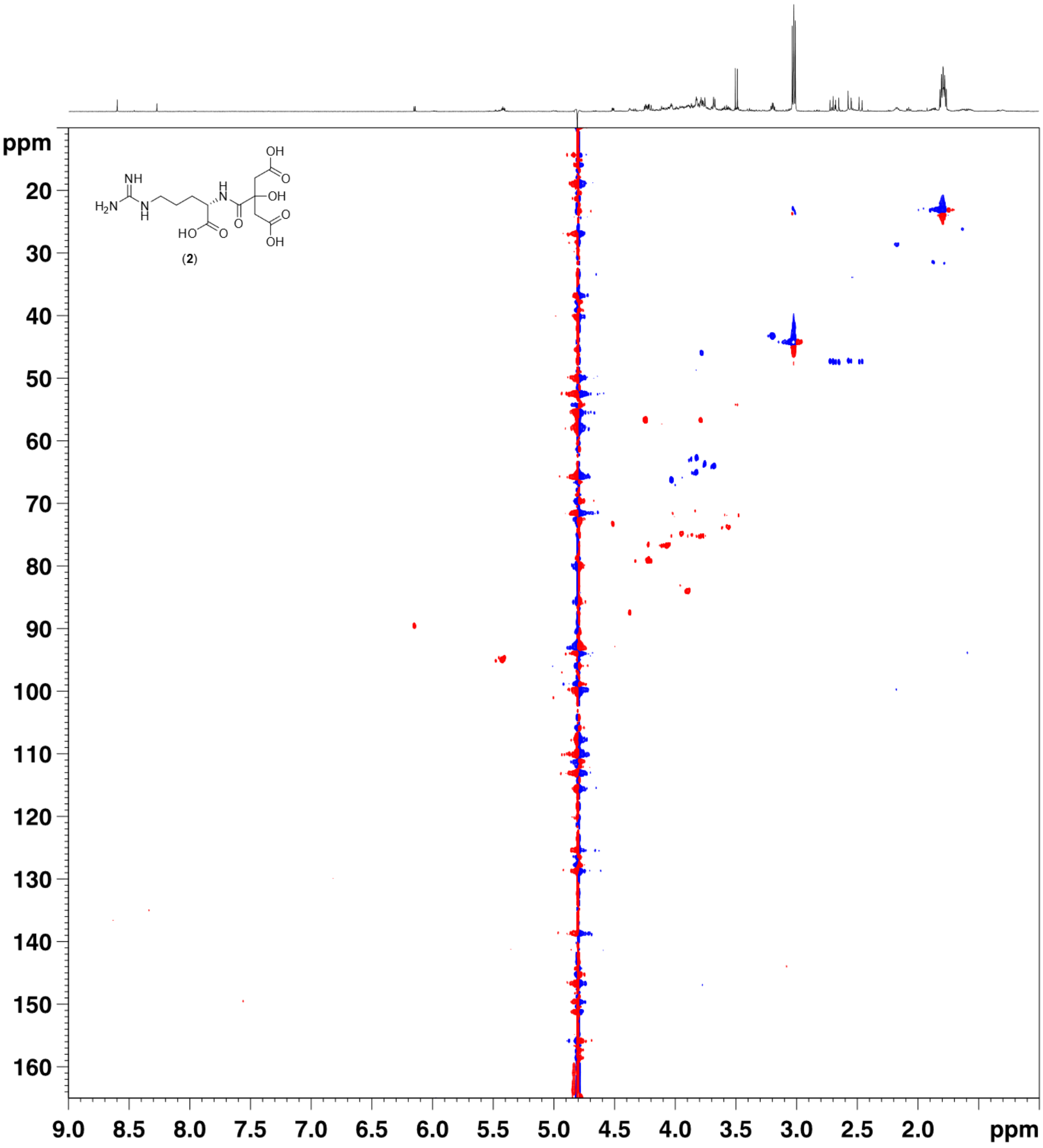

Figure S6: 2D HSQC spectrum of fraction 68 of the polar pine nut extract containing N^{α}-(2-hydroxy-2-carboxymethylsuccinyl)-L-arginine (**2**). The HSQC spectrum was acquired using the hsqcedetgpsisp2.2 pulse sequence on the Bruker Avance NEO 600 MHz NMR Spectrometer using TopSpin 4.1.3, equipped with a 5 mm TCI Cryoprobe cooled with liquid nitrogen. The spectrum was acquired with 2048 data points in F2 dimension and 128 increments using nonuniform sampling with NUSAmount of 50%. After acquisition of 16 dummy scans, 384 scans were acquired.

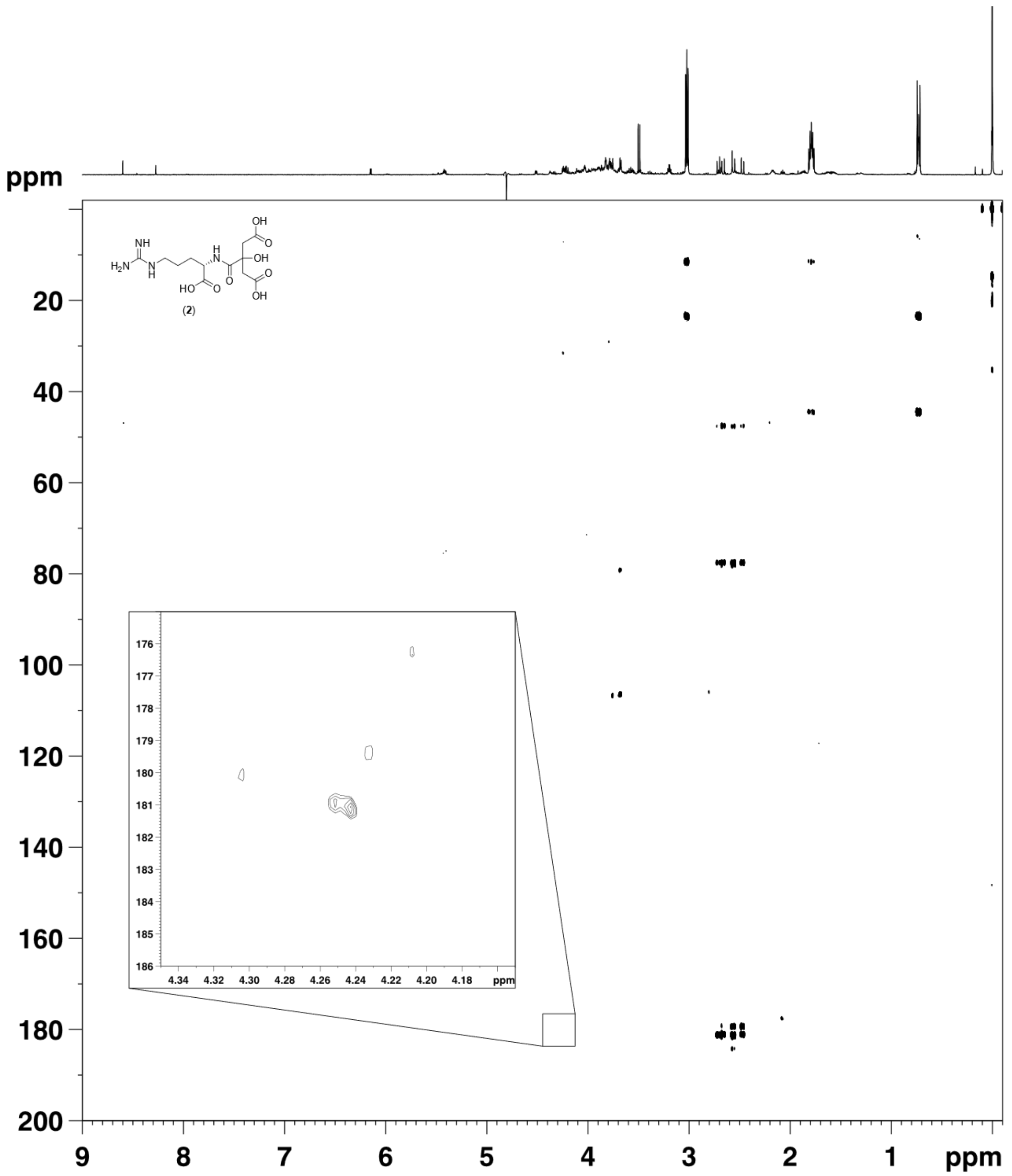

Figure S7: 2D HMBC spectrum of fraction 68 of the polar pine nut extract containing N^{α}-(2-hydroxy-2-carboxymethylsuccinyl)-L-arginine (**2**). The HMBC spectrum was acquired using the hmbcetgpl3nd pulse sequence on the Bruker Avance NEO 600 MHz NMR Spectrometer using TopSpin 4.1.3, equipped with a 5 mm TCI Cryoprobe cooled with liquid nitrogen. The spectrum was acquired with 4096 data points in F2 dimension and 256 increments using nonuniform sampling with NUSAmount of 50%. After acquisition of 32 dummy scans, 512 scans were acquired. The enlarged signal shows the correlation of H-2’ to C-1’ with a signal-to-noise ratio of 3.3.
